# Future urban development exacerbates coastal exposure in the Mediterranean

**DOI:** 10.1038/s41598-020-70928-9

**Published:** 2020-09-02

**Authors:** Claudia Wolff, Theodore Nikoletopoulos, Jochen Hinkel, Athanasios T. Vafeidis

**Affiliations:** 1 Coastal Risks and Sea-Level Rise Research Group, Department of Geography, Christian-Albrechts University Kiel, Kiel, Germany; 2 Unaffiliated, Athens, Greece; 3 Global Climate Forum E.V. (GCF), Berlin, Germany; 4 Division of Resource Economics, Thaer-Institute and Berlin Workshop in Institutional Analysis of Social-Ecological Systems (WINS), Humboldt-University, Berlin, Germany

**Keywords:** Environmental sciences, Natural hazards

## Abstract

Changes in the spatial patterns and rate of urban development will be one of the main determinants of future coastal flood risk. Existing spatial projections of urban extent are, however, often available at coarse spatial resolutions, local geographical scales or for short time horizons, which limits their suitability for broad-scale coastal flood impact assessments. Here, we present a new set of spatially explicit projections of urban extent for ten countries in the Mediterranean, consistent with the Shared Socioeconomic Pathways (SSPs). To model plausible future urban development, we develop an Urban Change Model, which uses input variables such as elevation, population density or road network and an artificial neural network to project urban development on a regional scale. The developed future projections for the five SSPs indicate that accounting for the spatial patterns of urban development can lead to significant differences in the assessment of future coastal urban exposure. The increase in exposure in the Extended Low Elevation Coastal Zone (E-LECZ = area below 20 m of elevation) until 2100 can vary, by up to 104%, depending on the urban development scenario chosen. This finding highlights that accounting for urban development in long-term adaptation planning, e.g. in the form of land-use planning, can be an effective measure for reducing future coastal flood risk on a regional scale.

## Introduction

The urban extent in low lying coastal areas is increasing faster than in other regions^1^, thus leading to increased exposure to sea-level rise and associated hazards. Societies' risk from these hazards will, therefore, not only depend on the physical drivers of change but also on the rate and pattern of urban growth which will be guided, to a large extent, by policies on future urban development^2^. One way to investigate how urban development influences future coastal flood risk is by accounting for spatiotemporal urban land cover change with the use of spatially explicit future urban projections in coastal impact assessments. Spatially explicit future urban extent scenarios are, however, currently often not available on a regional scale, which is one of the major shortcomings in coastal impact assessments to date^3^. Until recently, most studies have considered physical drivers of future change, partly accounting for population and economic development^4^ but have neglected changes in the spatial extent of urban agglomerations, where most impacts occur. Large scale urban change models can help to better understand how a non-climatic driver, namely urban development, influences future risk.

Different concepts and methods have been developed for modelling future urban change. Consequently, there now exists a vast diversity in modelling approaches, concepts, and models striving to describe and understand the mechanisms of land cover change. These approaches include, among others, the use of Cellular Automata^5^, Monte Carlo simulations^6^, Artificial Neural Networks^7^, as well as process-based and empirical models (e.g. CLUE)^8^. To date, most spatially explicit urban extent studies involve local applications^2,7,9,10^, and few broad-scale studies exist. Examples of regional-scale applications of urban change models include the studies of Seto et al.^11^, Zhou et al.^12^ and Gao and O'Neil^13^. Seto et al.^11^ used the land change model GEOMOD and a Monte Carlo simulation approach using projected GDP and urban population to develop spatially explicit probabilistic forecasts of urban land cover change until 2030 with a resolution of ~ 5 km. Zhou et al.^12^ used the SLEUTH Urban Growth model to develop urban land cover projections between 2012 and 2050 based on urban maps generated from LandScan population data at a resolution of 1 km. SLEUTH is an abbreviation that stands for all the inputs used in the model, which are slope, land cover, excluded regions, urban land cover, transportation and hill shade (Zhou et al. 2019). Recently, Gao and O'Neil^13^ developed an empirical model based on spatial data, the Spatially Explicit Long-term Empirical City developmenT (SELECT) model, to project urban land cover over the 21 century with a resolution of ~ 14 km. All the studies mentioned above are too coarse, in terms of spatial resolution, for regional coastal applications while in most cases the time horizon of the projections is too short for long-term coastal impact assessments.

In this paper, we address this gap by developing spatially explicit projections of urban extent for 10 Mediterranean countries with a resolution of 100 m, which is 10 to 140 times finer than existing gridded urban extent projections. For this purpose, we couple a Multilayer Perceptron (MLP) with a Geographic Information System to generate spatially explicit urban extent projections, for a range of socio-economic scenarios, until 2100. MLP is one of the modelling tools that has recently been used in the attempt to explore the complexity of interactions that govern the patterns in which urban extent changes and evolve^14,15^. An MLP has the ability to quantify complex behaviour and patterns^16^ by taking into account non-linear relationships between the input and output variables and generalise in the presence of noisy or incomplete data^17^.

Our projections are quantitatively and qualitatively consistent with the assumptions of the global Shared Socioeconomic Pathways (SSPs), which have been developed to support vulnerability and impact assessment studies. The SSPs comprise narratives that describe five different plausible pathways of societal development and also include information on future urban outcomes (see Table 1)^18,19^. In specific, urbanisation in SSP1 and SSP5 is rapid, although it is less well managed in SSP5^19^, leading to higher urban sprawl. In SSP3 urbanisation proceeds slowly with mixed spatial patterns, particularly in developing countries, which are characterised by inequality and fragmentation. Urban settlements are poorly planned, and limited urban employment opportunities lead to unattractive urban centres and slow urbanisation rates in SSP3. However, population growth is rapid under SSP3^20^ (except for the wealthier OECD countries) leading to high demand for urban space. A rapid urbanisation rate characterises SSP4 in medium and low-income countries, and central urbanisation rate in high-income countries with mixed spatial patterns, mainly driven by a lack of rural employment opportunities. Population growth is low in Mediterranean countries. SSP2 describes a world with a development that occurs at rates consistent with historical patterns of urbanisation and spatial patterns^19^.

**Table 1:** Summary of the Shared Socioeconomic Pathway assumptions underlying urban development (based on Jones and O'Neill^19^; Jiang and O'Neill^18^ and KC and Lutz^20^).

	Urbanisation rate	Urban spatial patterns	Population growth	GDP growth
SSP 1	Rapid	Urban expansion well managed (compact)	Global population growth is relatively low; fertility is medium in wealthier OECD countries	High-income growth, but slower economic growth over the longer term
SSP 2	Central	Historical spatial patterns	Global population growth is moderate and levels off in the second half of the century	Medium income growth
SSP 3	Slow	Mixed urban spatial patterns	Population growth is rapid (except for the wealthier OECD countries) leading to high demand for urban space	Low-income growth
SSP 4	Rapid in medium and low-income countries, central in high-income countries	Mixed spatial patterns	Population growth is low in Mediterranean countries	Low-income growth
SSP 5	Rapid	High urban sprawl, urban expansion is not well managed (compared to SSP1)	Population growth is high in wealthier OECD countries, low population growth in developing countries	Rapid growth of the global economy

In a final step, we use the developed scenarios to investigate how coastal exposure changes in the future. We choose the Mediterranean region as it is considered a hotspot of urban development globally. Between 1960 and 2010, the urban population increased by 20%^21^. A large share of urban development takes place along the coast where most of the industry and services are located^22^. According to Seto et al.^11^, the region is expected to experience a 160% increase in urban extent between 2000 and 2030. Therefore, many Mediterranean cities will be potentially exposed to climate-related hazards such as coastal flooding and erosion. Even though the current level of risk is not high in the Mediterranean, it is likely to increase in the future^23,24^, also due to socio-economic development. Thus, the Mediterranean region can be considered as an adaptation hotspot^25^.

## Results

### Spatially explicit projections of urban extent

**Figure 1:**
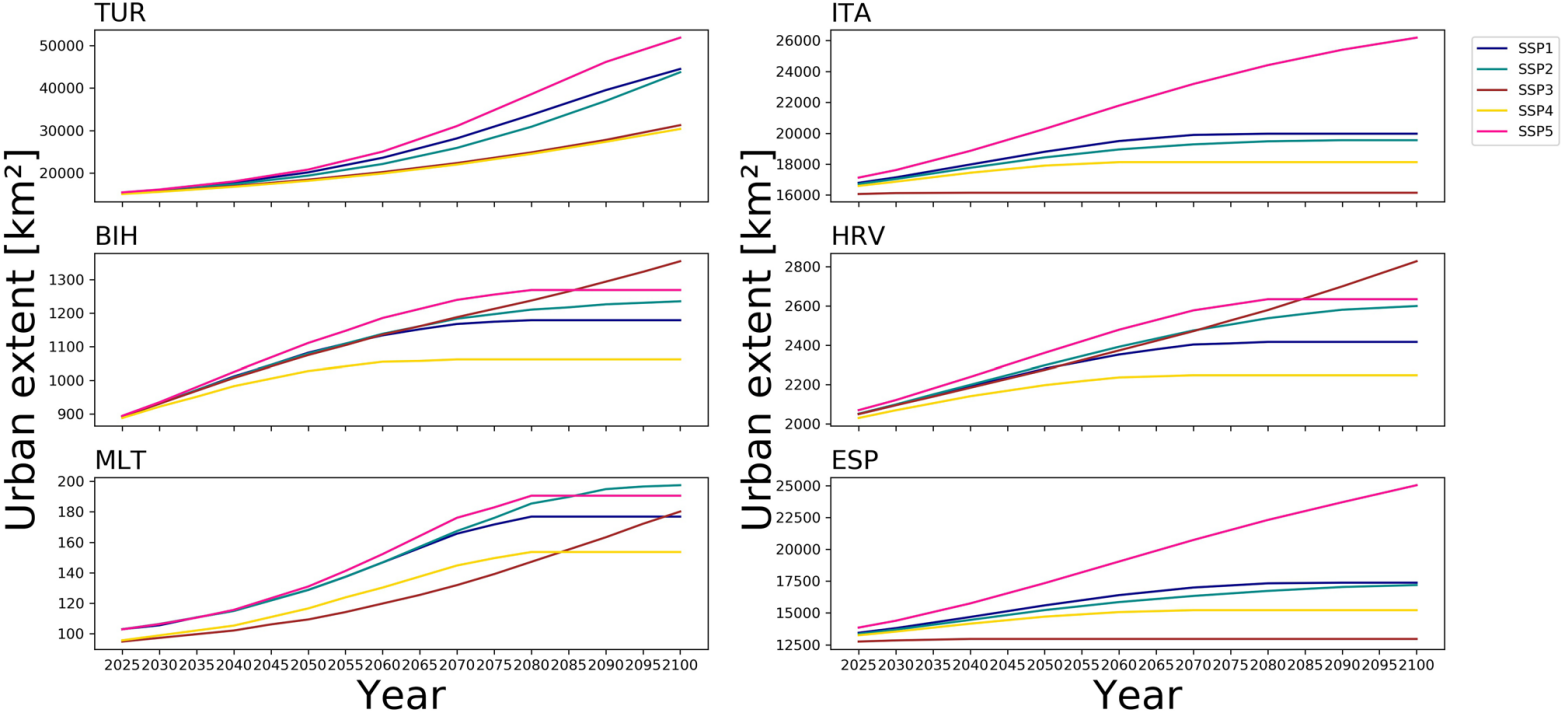
Temporal development of urban extent for six selected countries from 2025 to 2100. Note that the scales on the y-axis are different for each graph *(created with Python 3.7.4 using matplotlib,* https://www.python.org/downloads/release/python-374/).

**Figure 2:**
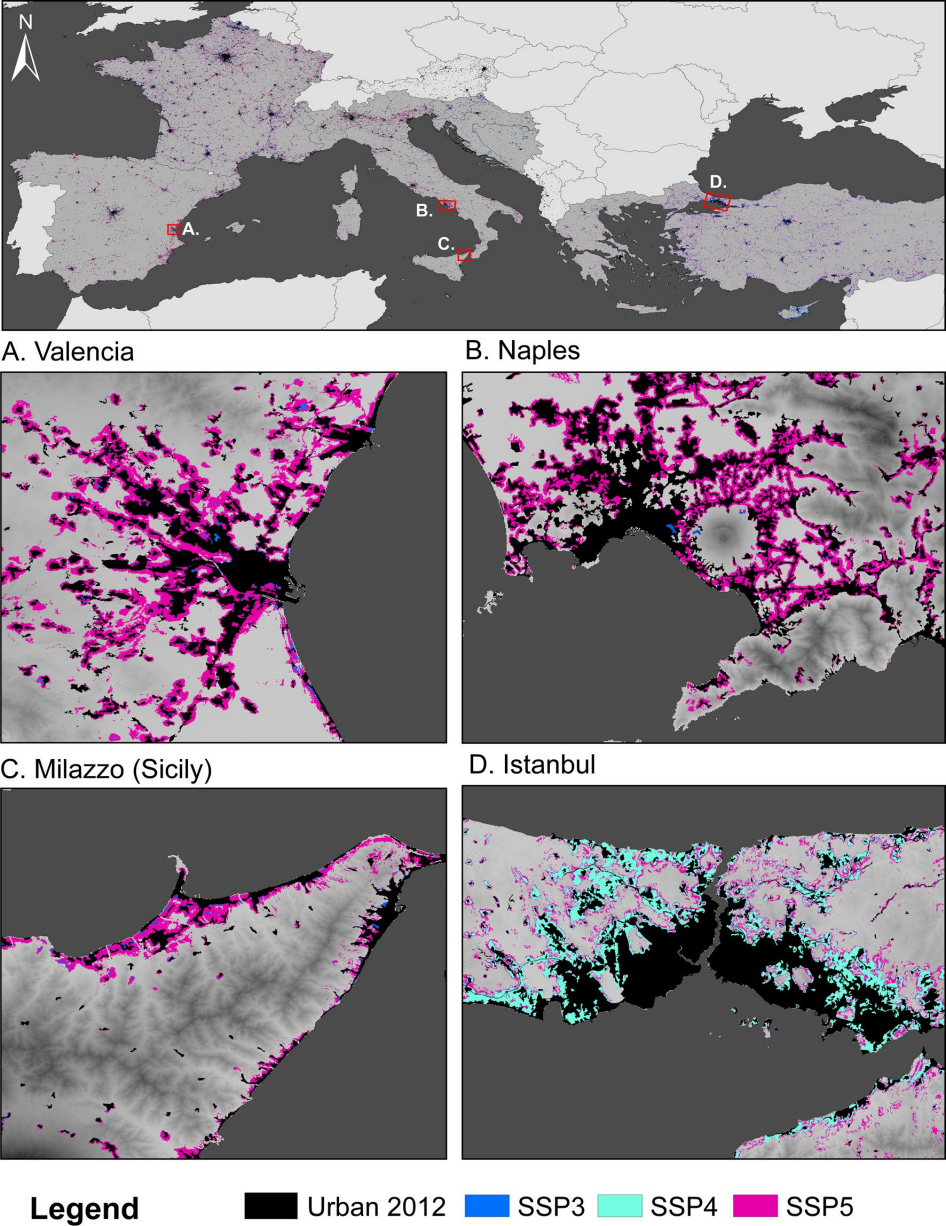
Spatially explicit urban extent in 2100 for different SSPs. European Mediterranean Countries included in the study are highlighted in darker shades of grey in the upper panel. For four selected regions, we present the spatially explicit urban extent scenarios that produce the highest and lowest urban extent. (Maps *created with ArcGIS 10.6.1*)

At the Mediterranean scale, we find substantial differences in future urban development rates and patterns until 2100 between the different projections (Figs. 1 and 2). The difference between the high and low urban extent projection ranges between 25% (HRV) and 115% (FRA) within the countries in the year 2100. Under all SSPs, urban extent increases during the century (Fig. 1). Until 2085, SSP5 produces the highest urban extent in all countries, reflecting the underlying narrative of a high urbanisation rate combined with urban sprawl. Interestingly, for some countries, the urban extent under SSP3 is, towards the end of the century, higher than under SSP5 (see Fig. 1—HRV and BIH). Rapid population growth in lower-income countries seems to be the main driver for urban land demand after 2085. SSP3 is characterised by a low fertility rate and declining population in most of the developed world^20^, leading to limited growth in urban extent in the wealthier European Mediterranean OECD countries (France, Greece, Italy, Slovenia, Spain) as well as in Turkey and Malta until 2100. In contrast, in the same scenario, the urban extent is highest in Bosnia-Herzegovina, Croatia, and Cyprus due to rapid population growth in those countries under SSP3. SSP5 shows the highest urban extent in wealthier European Mediterranean OECD countries and second highest in the rest of the countries (except for Malta). SSP5 is characterised by a high population growth driven by optimistic economic outlooks leading to a high GDP in high-income countries^26^. At the same time, in the rest of the countries, rapid development causes slower population growth compared to SSP3^19^. SSP1 is characterised by fast urbanisation rates and concentrated spatial patterns^18^ in all countries but a relatively low population increase. This leads to the second-highest urban extent in the wealthier European Mediterranean OECD countries and Turkey. In contrast, the increase in urban extent is relatively low in Malta, Bosnia-Herzegovina, Cyprus, and Croatia, where the population seems to be the primary driver of future urban extent in 2100. In all countries (except for Malta) SSP2 represents a middle-of-the-road urban future with moderate urban development (Fig. 1 and Supplementary Table 1 and Supplementary Fig. 1).

Ranking the SSPs according to their total urban extent in 2100 from lowest to highest (see Supplementary Table 2) shows that the wealthier European Mediterranean OECD countries follow the same patterns, with SSP3 having the smallest extent and SSP5 the highest. Bosnia-Herzegovina, Cyprus, and Croatia form a second group with similar patterns, this time with SSP4 having the lowest urban extent and SPP5 the highest, leading to up to 28% differences in the urban extent between the scenarios in 2100. Turkey and Malta seem to develop differently. Malta is highly urbanised, thus having the highest urban share of all countries (one-third of the nation is urban). The greatest urban extent for Malta occurs under SSP2, reflecting the historical pattern of urbanisation. Malta's high historical urban development rates combined with a high GDP under SSP2 lead to the highest urban extent. The high urban share in Malta leads to very few rural pixels that can convert in the future to urban. We investigated that the spatial allocation of future urban land may be plausible but somewhat unrealistic in Malta. We argue that such a high urban development rate in such a small country illustrates the challenges of urban land allocation in the future if urban development will be rapid and not well managed.

### Future coastal urban exposure in 2100

**Figure 3:**
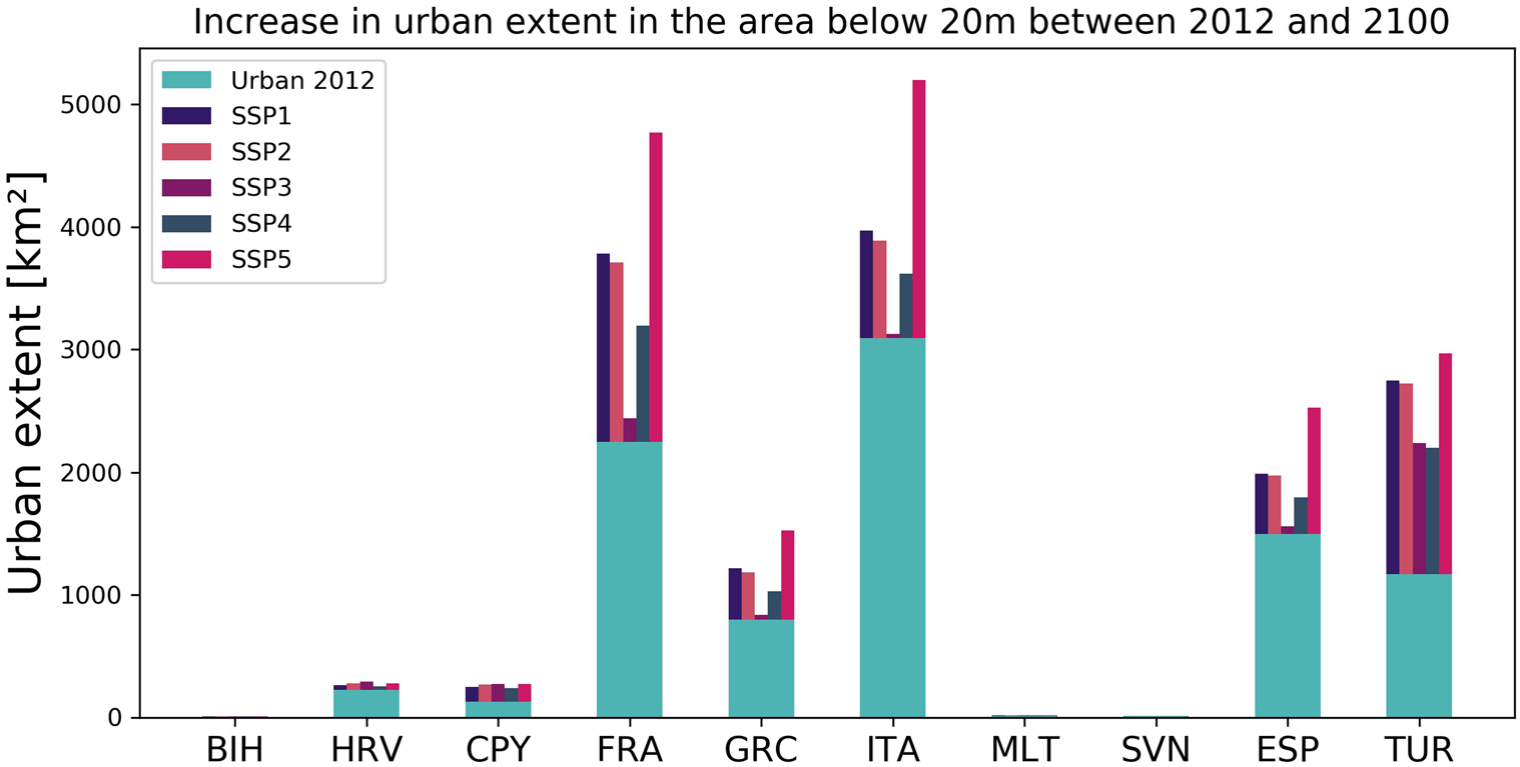
Country-specific increase in urban extent in the E-LECZ between 2012 and 2100 [in km^2^] for the five SSPs (*created with Python 3.7.4 using matplotlib*, https://www.python.org/downloads/release/python-374/).

Depending on the urban development scenario chosen the urban extent increases in the E-LECZ (area below 20 m) between 2012 and 2100, for instance, by 67% (2,075 km^2^) for Italy, 104% (2,331 km^2^) for France (Mediterranean coast only) and 86% (691 km^2^) for Greece (Fig. 3 and Supplementary Table 3).

**Figure 4:**
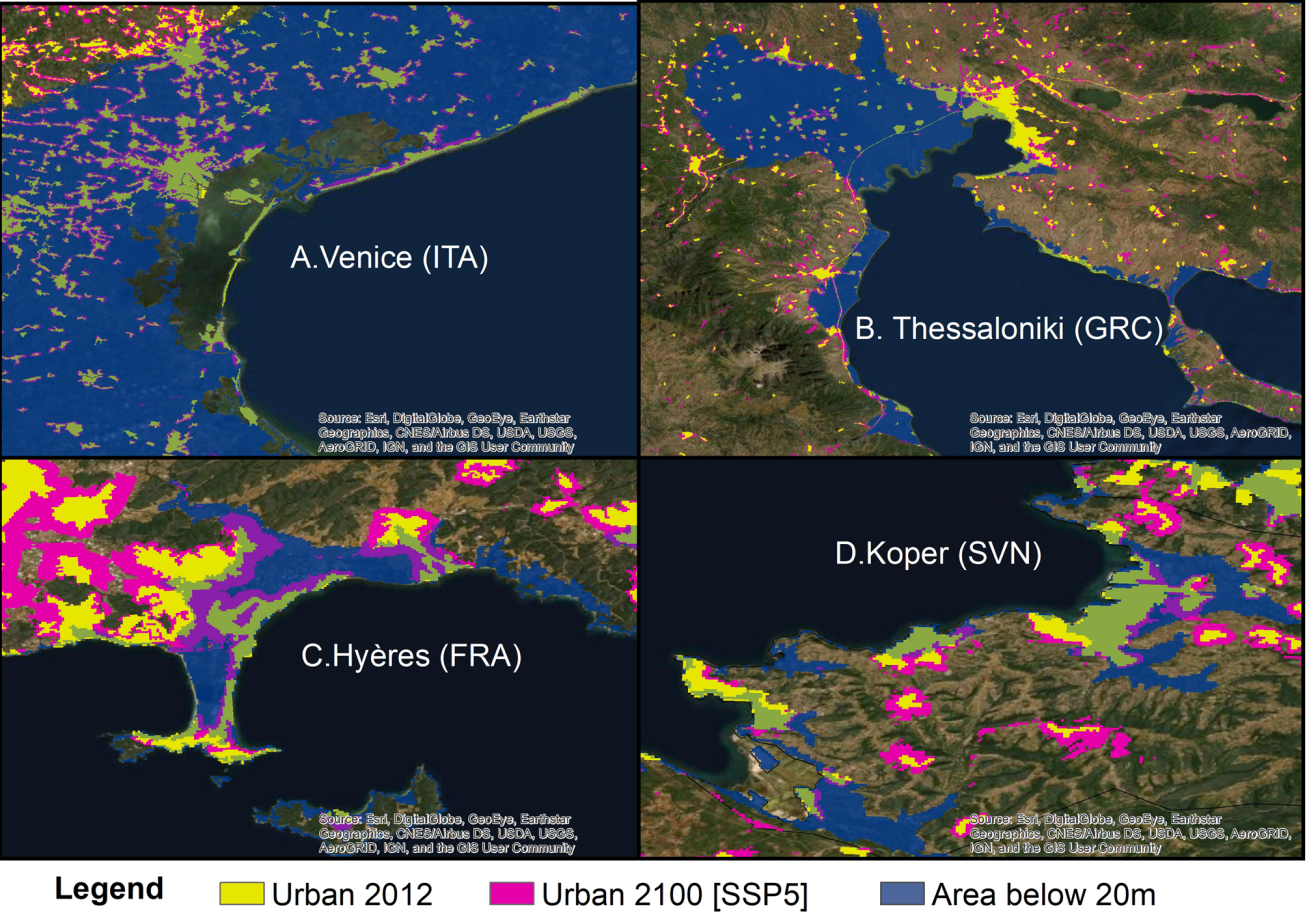
Potential urban development in four selected coastal regions under SSP5. In dark blue, the area that lies below 20 m is indicated (*created with ArcGIS 10.6.1*).

The urban extent scenario that leads to the highest urban exposure in the E-LECZ is the one that also produces the highest total urban extent in 2100 (see section "Spatially explicit projections of urban extent") reflecting that the model simulates high urban development in the E-LECZ. Figure 4 shows the spatial difference between 2012 and 2100 for four selected regions under SSP5. Compared to the year 2012, the urban extent increases in the floodplain in all regions leading to a significant increase in coastal exposure. Our results, therefore, show that accounting for the spatial patterns of coastal development can lead to significant differences in future exposure.

## Discussion

The ability to project future urban development enables researchers and policymakers to better anticipate the consequences of specific actions, e.g. in the context of urban planning^2^**,** or to plan appropriate future inventions that can reduce exposure to future climate-related hazards such as coastal flooding. The spatially explicit urban extent scenarios offer, therefore, the possibility to analyse different urban futures and designate future priority areas for coastal adaptation in the Mediterranean. In this context, the results of this study can inform the development of future coastal management and adaptation policies on a regional scale^27^, such as those outlined in the Integrated Coastal Zone Management (ICZM) protocol of the Barcelona Convention^28^.

Our study shows that one of the most effective measures to reduce future exposure will be to reduce future urban development inside the coastal floodplain. The ICZM Protocol prescribes the implementation of a 100 m setback zone that restricts further development within this zone^29^. Spatial planning in the form of setback zones or even retreat can be one of the most effective interventions to reduce future exposure^30^ and lead to a more sustainable Mediterranean future urban development. However, coastal urban development could also entail positive effects to reduce future vulnerability to natural hazards and hence increase the overall adaptive capacity of cities^31^. Even if future urban exposure increases, the coastal vulnerability could decrease if actual adaptation action (e.g. in the form of hard protection) and disaster management are effectively implemented. Hence, we argue that the concentration of people and assets could also increase the efficiency and effectiveness of adaptation measures in the future. Future urban extent scenarios could be useful to explore these avenues and inform adaptation and spatial planning authorities what mix of adaptation strategies for different regions could be most effective.

Several studies of European cities had shown that even when population decreased urban extent increased^32,33^. This trend is also reflected in our developed spatially explicit urban extent scenarios, where urban extent increases with time under all scenarios, albeit at lower rates in those cases where population declines. The urban projections reflect that urban development is linked to GDP growth in high-income countries. In contrast, in lower-income countries, urban development is more driven by population growth towards the end of the century. This pattern has also been reported in other studies (e.g. see meta-analysis of global urban land expansion from Seto et al. 2011^1^).

### Model performance and limitations

Evaluating our country-specific urban change models against historical data indicates that these are capable of reproducing historical spatial patterns of urban development on a regional scale. We observed that the models of those countries with a high urban share and/or uniform urban sprawl patterns (see Table 2), such as Croatia, Slovenia, France or Cyprus, perform best. This indicates that the MLP model can better generalise on countries where a large enough sample of urban change is provided. As the extent of urban areas is small in comparison to that of rural areas, we have an imbalanced dataset due to an uneven distribution of classes (much more rural pixel, than urban). This poses a challenge from a Machine Learning perspective since models are harder to train with imbalanced training sets. This issue has been tackled by under-sampling the majority that is the rural class in our case, to create a more balanced training set. For most countries, the model performed best with an under-sampling ratio of 10 (1:10, Urban: Rural) (the undersampling ratio used for every country can be found in Supplementary Table 4). Further, we improved the performance of the MLP model by using data augmenting which creates additional training data. At the same time, the MLP is challenged when trying to reproduce inhomogeneous settlements, where urban development is fragmented with clustered and contrasted patterns. Such an example is Spain, where the performance of the MLP was the lowest (Table 2).

**Table 2:** Performance measures of the MLP for predicting urban extent in 2012 for every country.

country	Overall accuracy	Precision	Recall	Negative predictive value	Specificity	F1-score
BIH	0.997	0.901	0.888	0.998	0.998	0.894
CYP	0.992	0.955	0.951	0.995	0.996	0.953
ESP	0.986	0.745	0.635	0.991	0.995	0.680
FRA	0.992	0.967	0.892	0.994	0.998	0.928
GRC	0.993	0.893	0.852	0.996	0.997	0.871
HRV	0.996	0.950	0.937	0.998	0.998	0.939
ITA	0.993	0.970	0.890	0.994	0.998	0.927
MLT	0.999	0.999	0.999	0.999	0.999	0.999
SVN	0.999	0.980	0.977	0.999	0.999	0.978
TUR	0.992	0.788	0.731	0.995	0.996	0.759

The explanatory potential of the input variables was analysed by using entropy and mutual information^34^. Mutual information measures the dependencies between each feature and the output. It quantifies the information content of two variables in classification problems by considering the input–output statistics without any reference to a specific model. Mutual information should be interpreted relative to the entropy. The mathematical explanations of the two metrics can be found in the supplementary material. Supplementary Table 5 shows the mutual information and entropy values for every input variables and country. High mutual information means that the input feature is suitable in explaining the output. In nearly all countries, distance to urban land cover and the population density have the highest mutual information, reflecting the fact that new urban development is expected to grow near existing urban agglomerations. However, the order of magnitude between the variables is often similar, thus we believe that all input variables are useful for our MLP (see Supplementary Table 6). The entropy of a country can be interpreted as a measure of urbanisation in this study. In countries where the distribution of urban and rural pixels is more balanced (e.g. MLT 29% Urban; 70% Rural), the entropy is high (for MLT 0.8).

We must note that the validation measure' overall accuracy' along does not necessarily mean that the urban change model has high predictive power. Pontius and Malanson^35^ discuss several reasons why 'percent correct' alone is not enough to validate a model. Importantly, they state that a null model that predicts pure persistence between two-time steps often leads to more than 90% correct due to the temporal autocorrelation between the time steps. Additionally, the merit is not appropriate for imbalanced datasets^36^. Therefore, it is essential to consider the f1-score, precision, recall and a visual assessment of the spatial performance of the urban change model to assess the performance.

Future research could improve and extend this work in a number of ways. First, in our study, we assume a constant pattern of the input variables throughout the century due to lack of data. Hence, one central assumption of the study is that similar processes drive future urban development. For instance, urban development will change as a response to a changing road network but information on the future development of the road network is currently not available for most countries. The MLP model would be able to incorporate such processes if input data for all variables become available in the future, which would allow us to dynamically model future urban expansion from one time step to another. Second, the developed urban change model only considers spatial aspects and does not account for non-spatial elements such as demographic characteristics, spatial planning policies or tipping points that are unpredictable (such as natural hazards) which is a major challenge in urban land change models^16^. Future work could further expand the urban scenarios by accounting for policy interventions such as coastal adaptation measures in the form of setback zones that restricts future urban development in flood-prone areas or include vertical growth of cities into the scenarios. Last, a time interval of more than 12 years between the input and target data could lead to improved training of the MLP by providing a larger number of urban pixels that change from one time step to another. However, the lack of a consistent land cover time series with a high resolution that can be used as input (e.g. distance to forest, grassland) and output data (urban) to train the MLP is currently not available at this scale.

## Conclusion

Urban growth rates and spatial patterns of urban development will determine society's exposure and vulnerability to extreme events, sea level, and other climate-related hazards. Urban development is a dynamic and complex process^37^ that is influenced by physical, socio-economic and political conditions. Hence, how urban development influences future risk has not yet been quantified and is not well understood^2^. Spatiotemporal land cover analysis and future urban land cover projections can help improve our understanding of these processes.

We have produced urban extent projections that cover a plausible range of uncertainty with a resolution of 100 m, spanning from 2025 to 2100. The developed urban projections are based on the global SSPs and thus depict five different plausible urban development scenarios. Across all SSPs, we observe a continued increase in urban areas in the coastal zone, which leads to an increase in coastal exposure, even though the rates and spatial patterns of urban development vary between the different scenarios. We anticipate that the developed urban extent scenarios will be employed in a wide range of impact assessments to climate change-related hazards beyond coastal applications.

## Methods

**Figure 5:**
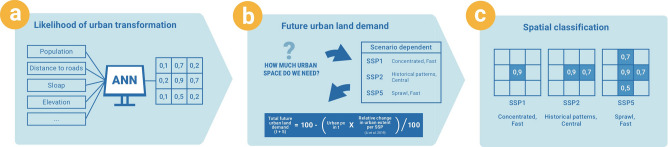
Workflow of the urban change model *(created using Photoshop CS4*).

To model plausible future urban extent, we developed a set of country-specific urban change models (see Fig. 5) as the rate and pattern of urban development can vary considerably across countries and years^38^. First, we employ an MLP artificial neural network for modelling the likelihood of urban transformation (see Fig. 5a). In a second step, we calculate, for each SSP, the future urban land demand in 5-year time steps until 2100 (see Fig. 5b). Finally, we create the urban extent scenarios by classifying the MLP model outputs accordingly (see Fig. 5c). These projections are then employed for calculating future exposure to coastal flooding (section “Future coastal flood exposure”).

### Urban change model

#### Step a: Likelihood of urban transformation

We used an MLP to generate a predictive spatially explicit model of urban development for 10 Mediterranean countries where data were available (MLT, CYP, GRC, SVN, ITA, BIH, HRV, FRA, ESP, TUR). An MLP is a computational model often used for complex, non-linear behavior, and patterns of unknown input–output relations^7,39^. Every MLP is composed of one input layer, one or several hidden layers, and one output layer. For a detailed description of MLPs see reference^34^.

The MLP was trained using input data from 2000 to reproduce CORINE urban land cover data from 2012 (https://land.copernicus.eu/). In this study, we define urban areas as locations dominated by artificial surfaces, which corresponds to the artificial surface class (code 1) from CORINE that includes residential, industrial, commercial, transport and green urban areas.

The input layer of our MLP consists of nine input variables (Table 3). The input variables have been selected based on literature review and data availability. Elevation and slope have been selected to reflect the topography, which influences urban development as steep areas, and areas of high elevation are less likely to develop^7^. Distance to roads has been included because areas that are easy to access are more likely of being developed^40^ as it is correlated to people's mobility^7^, which influences where people decide to settle. As the study focuses mainly on the exposure of urban areas to sea-level rise and climate-related hazards such as storm surges, we decided to also include the distance to the coast as an input variable. Low elevation coastal zones are developing faster than the hinterland^38^ and therefore distance to the coast can be an important predictor. We also included the Euclidian distance to urban areas, arable land, grassland, and forest to account for the effect of other types of land use on urban development. Finally, we used population density as an input variable to reflect socio-economic driving forces, as numerous studies show that these constitute underlying drivers of urban development.

**Table 3:** Summary of data used for input and output variables of the MLP.

Variables		Source	Spatial resolution	Pre-processing notes
Input	Distance to forest	CORINE 2000	100 m	Corine classes used: 311, 312, 313, Euclidean distance to forest
	Distance to grassland	CORINE 2000	100 m	Corine classes used: 321, 322, 323, 324, Euclidean distance to grassland
	Distance to urban	CORINE 2000	100 m	Corine classes used: 111, 112, 121, 122, 123, 124, 131, 132, 133, 141, 142, Euclidean distance to urban 2000
	Distance to arable land	CORINE 2000	100 m	Corine classes used: 211, 212, 213, 221, 222, 223, 231, 241, 242, 243, 244, Euclidean distance to arable land 2000
	Distance to roads	Open Street Map		We used the main roads (fclass: primary motorways, secondary), Euclidean distance to roads
	Population density	GPW v4 2000	1 km	Total population in 2000 per km^2^, resampled to 100 m
	Elevation	SRTM	30 m	Elevation in m, resampled to 100 m
	Slope	Derived from SRTM	30 m	Percent increase in slope, resampled to 100 m
	Distance to coast	Based on CORINE coastline	100 m	Euclidean distance to coastline
Output	Urban	CORINE 2012	100 m	Reclassification of CORINE data to Urban = 1, Rural = 0; Corine classes used: 111, 112, 121, 122, 123, 124, 131, 132, 133, 141, 142

The output layer consists of a single variable giving the likelihood that a pixel will turn urban in the next time step. To define the best MLP architecture for every country (i.e. the number of neurons in each layer, the connection patterns between the layers, the activation function and the learning methods) we conducted a sensitivity analysis using between 6 and 10 different network architectures. An overview of the best model architecture per country can be found in Supplementary Table 4.

#### Step b: Future urban land demand

In the second step, we calculated the future urban land demand for every country, SSP and time step. For this purpose, we used the relative changes in total urban extent for every country between 2020 and 2100, obtained from Li et al.^38^^.^ This study developed (non-spatial) country‐specific urban extent growth models using data from an urban extent time series, spanning from 1992 to 2013 (22 years) based on satellite observations, and historical socio-economic indicators, namely Population and GDP. These country-specific models were then used to project future urban growth under the Shared Socioeconomic Pathways until the end of the twenty-first century using the population and GDP data from the SSP database. The reported mean accuracy for Europe compared to fine resolution land cover data was 95%^38^. In our model, we used the relative changes of Li et al. 2019 to calculate the total number of future urban grid cells.

#### Step c: Spatial classification

Finally, the future urban land demand per time step was used to reclassify our MLP outputs. The highest urban transformation likelihoods were reclassified according to the total number of future urban grid cells (see Fig. 5). We used CORINE 2012 as a starting year to create high-resolution spatially explicit urban extent scenarios that are consistent with the global SSP assumptions, in 5-year time steps until 2100. We produced scenarios for 10 European Mediterranean countries where global SSP assumption and CORINE data exist.

### Future coastal flood exposure

Using the spatially explicit urban extent projections, we then assessed future coastal exposure for the Mediterranean coast in 2100. For this purpose, we calculated the low-lying part of the coastal zone that is hydrologically connected to the sea and lies above 20 m of mean sea level (E-LECZ). We decided to use the E-LECZ to account for all plausible future changes in mean sea level and associated extreme water levels^41^. The exposed area was overlaid with the developed urban extent projections to analyse the exposure of future urban extent for all SSPs.

### MLP performance

**Figure 6:**
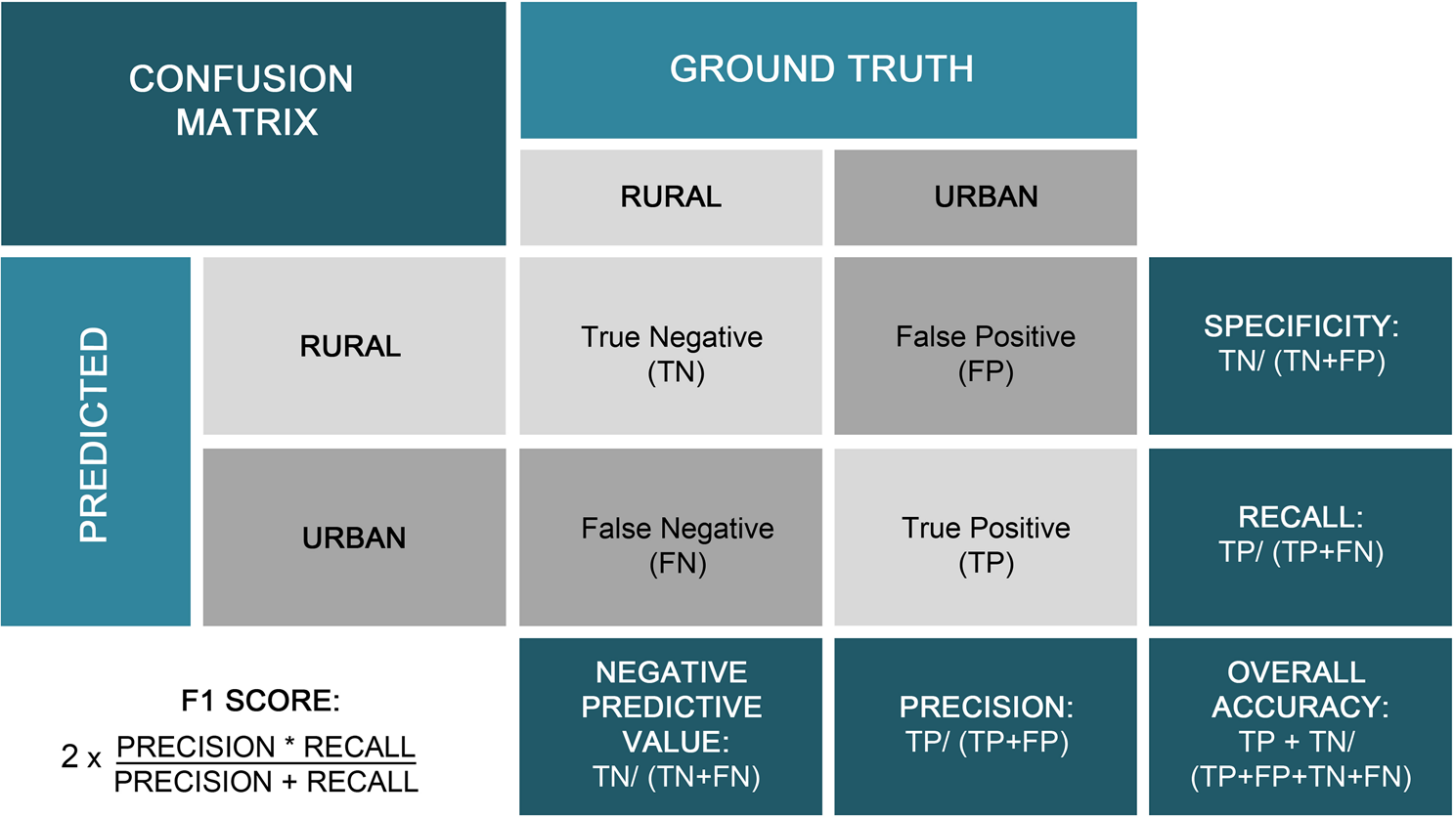
Confusion matrix *(created using Photoshop CS4*).

We used a confusion matrix to evaluate the model performance (see Fig. 6) and find the MLP model architecture that best approximates the input–output relationships. The confusion matrix is a way to investigate the percentage of correct and incorrect classified pixels and is a measure for analysing the goodness of fit for a specific classification model architecture. The overall accuracy reflects the overall correct classified pixels. However, this metric is very sensitive to imbalanced datasets and can be misleading when measuring the MLP performance^36^ as our dataset contains few urban pixels compared to the rural class. A more appropriate merit to assess the performance of binary classification problems with a skewed dataset is, therefore, the F1-score. The F1 score is used to represent the balance between precision (Positive predictive value—the percentage of pixels that are classified as urban and are, in reality, urban) and recall (True positive rate—the ratio of the pixels that are correctly classified in relation to the total positive (target) pixels). It can be viewed as the harmonic mean of precision and recall. The results per country are presented in Table 2.

The urban change models can reproduce the observed patterns of past urban development between 2000 and 2012 with an F1-score between 68 and 99% in all countries (see Table 2). This indicates that the country-specific urban change models capture past patterns of urban development. However, the confusion matrix is not very useful in assessing spatial patterns. For instance, if a pixel is misclassified, regardless of whether the correct class is found in a neighbouring pixel, it is presented as incorrect^35^. Therefore, a visual assessment was conducted by overlaying the simulated and CORINE 2012 maps using a Geographic Information System to explore the spatial and qualitative patterns of the predicted model outputs (see Supplementary Fig. 2 for an example).

## Supplementary information

Supplementary file1

## Data Availability

The urban extent projections are available at the figshare repository (https://figshare.com/s/8fc878cbf3597c6574e1). Further, we provide the python code to set up the MLP model and create the urban extent projection in the figshare repository.
