# Supplementary material for: Future urban development exacerbates coastal exposure in the Mediterranean
Source: Sci Rep. 2020 Sep 2;10:14420. doi: 10.1038/s41598-020-70928-9 (PMC7468119; doi:10.1038/s41598-020-70928-9)
Supplement: Supplementary file 1 — Supplementary file1 [file 41598_2020_70928_MOESM1_ESM.pdf]

Supplementary Information

Future urban development exacerbates coastal exposure in the Mediterranean

Claudia Wolff[1*], Theodore Nikoletopoulos[2], Jochen Hinkel[3,4], and Athanasios T. Vafeidis[1]

1 Coastal Risks and Sea-Level Rise Research Group, Department of Geography, Christian-Albrechts University Kiel, Kiel, Germany

2 Independent researcher

3 Global Climate Forum e.V. (GCF), Berlin, Germany

4 Division of Resource Economics, Thaer-Institute and Berlin Workshop in Institutional Analysis of Social-Ecological Systems (WINS), Humboldt-University, Berlin, Germany

*Corresponding Author: Claudia Wolff, e-mail: wolff@geographie.uni-kiel.de, tel: +49-431-880 1782

Supplementary Table 1: Country specific absolute urban extent in 2100 per SSP [in km²]

ISO	SSP1	SSP2	SSP3	SSP4	SSP5
BIH	1179	1235	1354	1062	1269
HRV	2416	2599	2828	2248	2634
CYP	2077	2338	2369	1900	2399
FRA	52005	50781	33269	42732	71523
GRC	5917	5723	4068	4957	7922
ITA	19970	19549	16146	18128	26190
MLT	177	197	180	154	191
SVN	1293	1210	1011	1156	1783
ESP	17375	17191	12956	15216	25028
TUR	44471	43701	31252	30366	51827

Supplementary Table 2: SSP ranging from lowest (left) to the highest (right) urban extent for all countries in 2100

ISO	Lowest urban extent	→			Highest urban extent
BIH	SSP4	SSP1	SSP2	SSP5	SSP3
HRV	SSP4	SSP1	SSP2	SSP5	SSP3
CYP	SSP4	SSP1	SSP2	SSP5	SSP3
FRA	SSP3	SSP4	SSP2	SSP1	SSP5
GRC	SSP3	SSP4	SSP2	SSP1	SSP5
SVN	SSP3	SSP4	SSP2	SSP1	SSP5
ESP	SSP3	SSP4	SSP2	SSP1	SSP5
ITA	SSP3	SSP4	SSP2	SSP1	SSP5
TUR	SSP4	SSP3	SSP2	SSP1	SSP5
MLT	SSP4	SSP1	SSP3	SSP5	SSP2

Supplementary Figure 1: Country-specific absolute urban extent [in km²] for the five SSPs in 2100. *created with Python 3.7.4 using matplotlib,* https://www.python.org/downloads/release/python-374/)

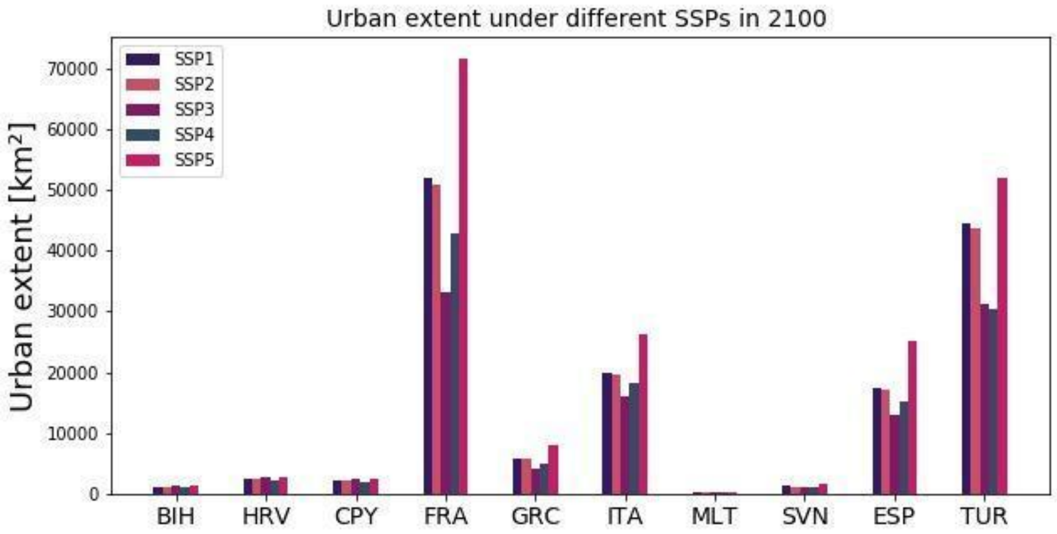

Supplementary Table 3: Country-specific absolute urban extent in the Mediterranean E-LECZ in 2012 and 2100 per SSP [in km²]

ISO	Base year 2012	SSP1 2100	SSP2 2100	SSP3 2100	SSP4 2100	SSP5 2100
**BIH**	4.3	7.3	7.6	8.1	6.6	7.8
**HRV**	224.6	263.6	276.0	290.0	250.7	278.2
**CYP**	129.1	250.3	269.0	271.3	236.8	273.9
**FRA**	2246.9	3780.3	3711.3	2439.8	3192.9	4770.2
**GRC**	799.1	1219.3	1183.4	836.2	1028.9	1528.1
**ITA**	3092.8	3968.7	3887.9	3124.1	3615.6	5199.5
**MLT**	11.7	16.3	19.0	16.4	14.8	17.5
**SVN**	11.0	12.7	12.5	12.0	12.3	14.1
**ESP**	1494.7	1990.0	1974.7	1561.3	1796.1	2530.8
**TUR**	1169.1	2746.9	2722.0	2240.0	2200.5	2968.8

Supplementary Figure 2: Spatial map of Athens showing the CORINE urban 2012 Land Cover data by the black outline. Colors show the likelihood of the MLP model. (*created with ArcGIS 10.6.1*)

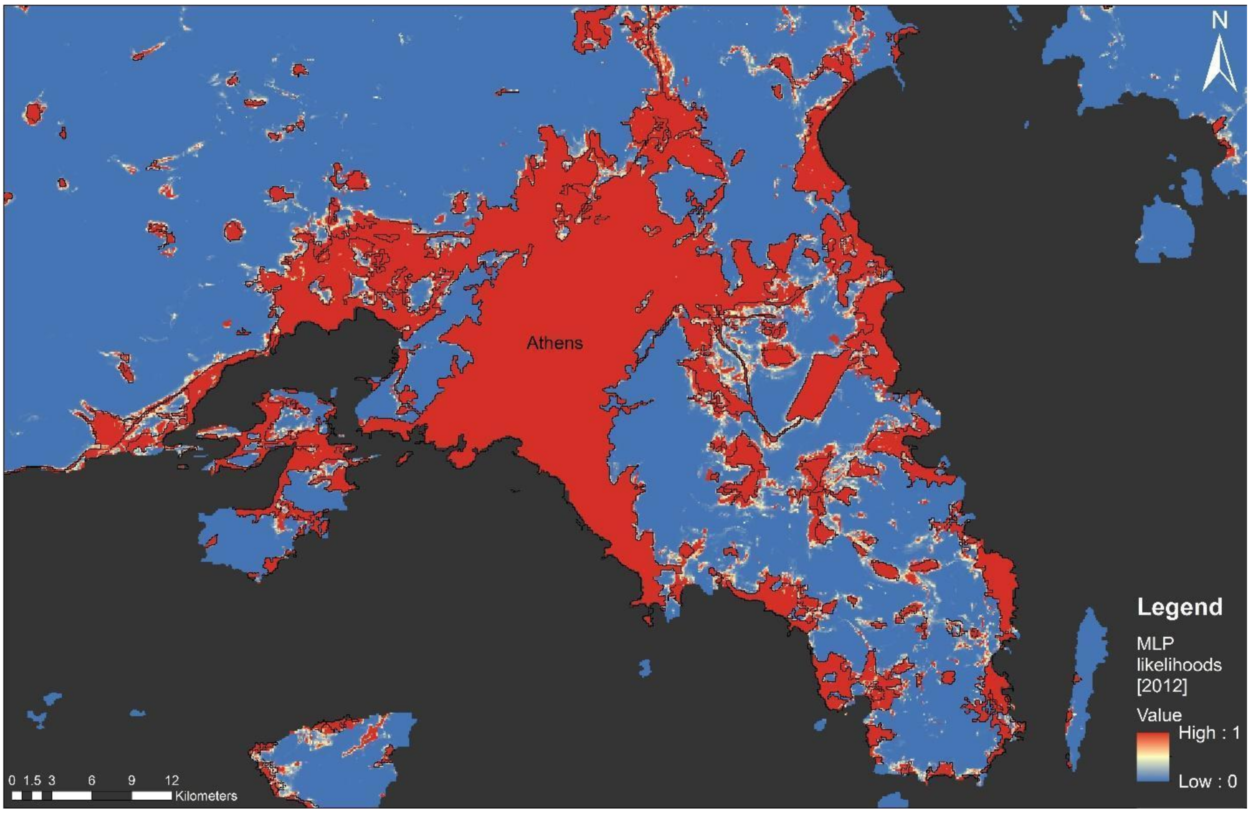

Supplementary Table 4: Best MLP model architecture per country

**Country**	**Hidden layer 1** [Nr of neurons]	**Hidden layer 2** [Nr of neurons]	**Hidden layer 3** [Nr of neurons]	**Hidden layer 4** [Nr of neurons]	**Activation function for the hidden layer**	**Under sampling factor**	**Activation function output layer**	**Optimization algorithm**
BIH	400	400	400	200	Relu	10	Sigmoid	Adam
CYP	400	400	200	-	Relu	10	Sigmoid	Adam
ESP	400	400	200	-	Relu	8	Sigmoid	Adam
FRA	400	400	400	200	Relu	10	Sigmoid	Adam
GRC	400	400	200	-	Relu	10	Sigmoid	Adam
HRV	400	400	400	200	Relu	8	Sigmoid	Adam
ITA	400	400	200	-	Relu	12	Sigmoid	Adam
MLT	200	200	200	100	Relu	False	Sigmoid	Adam
SVN	400	400	200	-	Relu	10	Sigmoid	Adam
TUR	400	400	400	200	Relu	10	sigmoid	Adam

Mutual Information and Entropy

The entropy can be calculated for a random variable Y by the following formula:

$$H(Y) = -\sum_{y} \mathbb{P}(y) \log_2(\mathbb{P}(y))$$

where P(y) is the probability function of the random variable Y, and log2 is the logarithm with base 2. Entropy is a measure of the uncertainty related to observing, or measuring the value of Y, which is only known prior to the observation with a certain probability. E.g. in a coin tossing experiment, we do not know beforehand the outcome (heads or tails), only the probability (50% if the coin is 'fair') that one of the will occur. The binary variable of a pixel in our study had the values urban or rural. The probability of a pixel being urban (p) (this can be calculated by counting the urban pixels and dividing by the total number of pixels), then the probability of a pixel being rural will be 1 − p. The entropy, in this case, is given (as a function of p) by:

$$H(p) = -p \log_2(p) - (1-p) \log_2(1-p)$$

This function has the following properties: It takes values in the interval [0, 1]. It attains its maximum H = 1 when the two events are equiprobable i.e. when p = 1/2. This is in agreement with the intuition that the uncertainty related to a binary random experiment is maximum when the two events have equal probability. On the other hand, if one of the two possible outcomes is known to occur with certainty (so actually we are not dealing with a random experiment), which in probabilistic terms would mean that p= 0 or p = 1, then the entropy is zero i.e. H = 0, reflecting the fact that there is no uncertainty about the outcome. The conclusion is that the more random an experiment is, the larger the uncertainty about the outcome as given by the entropy formula. The mutual information of two random variables X, Y is given by a similar formula,

$$I(X, Y) = \sum_{x,y} \mathbb{P}(x, y) \log_2 \left(\frac{\mathbb{P}(y|x)}{\mathbb{P}(y)} \right)$$

where P(x, y) is the joint probability function of the input-output pair X, Y, P(y|x) is the conditional probability of observing an output y given a particular value X = x of the input and P(y) is the probability function of the output. To understand what this formula says consider the two extremes: The output is independent of the input, i.e. the input does not have any effect on the output. This is expressed in probabilistic terms by P(y|x) = P(y). Then the argument of the log equals one, and the log equals zero resulting in zero mutual information. On the contrary, if by observing x it is certain that y will occur, P(y|x) = 1, and the mutual information equals the entropy of Y.

Supplementary Table 5: Mutual information and entropy per input variable and country

	ALB	BIH	CYP	ESP	FRA	GRC	HRV	ITA	MLT	SVN	TUR
**arable00**	0.01427702	0.01287891	0.03640366	0.03789122	0.07740804	0.01368984	0.0226428	0.05472601	0.29618769	0.01590871	0.04312946
**coast_dis**	0.0140481	0.0062767	0.01626422	0.01047858	0.00582252	0.00437606	0.010919	0.0113549	0.00255846	0.01531882	0.00348724
**forest00**	0.02096954	0.01798427	0.02070598	0.01312534	0.05965779	0.00904741	0.01805075	0.02398467	0.06937366	0.03231341	0.00435233
**gpw2000**	0.06426912	0.0322178	0.13166094	0.06121466	0.11313365	0.07435415	0.06244786	0.09974234	0.2670919	0.0747473	0.0357027
**grass00**	0.02097955	0.00447334	0.01608279	0.0285998	0.00763369	0.01712533	0.00898049	0.01782936	0.10541792	0.00309631	0.02707478
**road_dis**	0.02292437	0.01440244	0.0403217	0.02346158	0.06056939	0.02019458	0.03209766	0.04012055	0.03511463	0.0218958	0.01639844
**slope**	0.02106989	0.00830301	0.02442948	0.00496	0.00755278	0.01293146	0.0054379	0.0233271	0.02774585	0.01811359	0.00614875
**srtm**	0.02469795	0.00973831	0.02126464	0.01316451	0.01318467	0.01298798	0.01306852	0.02061855	0.02033104	0.01711588	0.00856534
**urban00**	0.06479063	0.0608467	0.21697443	0.06318127	0.19852776	0.08466114	0.11670051	0.17865163	0.5979203	0.1134459	0.05787689
**Entropy**	0.17814134	0.11699146	0.42558385	0.16309863	0.30851137	0.18860835	0.21010113	0.29880635	0.87387408	0.19164656	0.12838153

Supplementary Table 6: Mutual Information ranging (classification of the same order of magnitude) to show that all variables seem to be important input variables in modelling future urban extent

**Country**	**10^-1**	**10^-2**	**10^-3**
ALB		urban00, arable00, forest00, road_dis, coast_dis, srtm, gpw2000, grass00, slope	
BIH		arable00, forest00, gpw2000, road_dis, urban00	coast_dis, grass00, slope, srtm
CYP	urban00, gpw2000	arable00, forest00, road_dis, coast_dis, grass00, slope, srtm	
ESP		arable00, forest00, road_dis, coast_dis, grass00, srtm, urban00, gpw2000	slope
FRA	urban00, gpw2000	arable00, forest00, road_dis, srtm,	coast_dis, grass00, slope
GRC		arable00, road_dis, grass00, srtm, urban00, gpw2000, slope	coast_dis, forest00
HRV	urban00	arable00, forest00, road_dis, coast_dis, srtm, gpw2000	grass00, slope
ITA	urban00	arable00, forest00, road_dis, coast_dis, srtm, gpw2000, grass00, slope	
MLT	urban00, arable00, gpw2000, grass00	forest00, road_dis, srtm, slope	coast_dis
SVN	urban00	arable00, forest00, road_dis, coast_dis, srtm, gpw2000, slope	grass00
TUR		urban00, arable00, road_dis, gpw2000, grass00	coast_dis, forest00, srtm, slope
